# An Open‐Source, AI‐Supported Teaching Tool in Orthodontic Education—Assessment of Acceptance and Effectiveness

**DOI:** 10.1002/jdd.13916

**Published:** 2025-04-24

**Authors:** Hisham Sabbagh, Teodora Ribnishki, Linus Hötzel, Leonard von Bronk, Yeganeh Khazaei, Andrea Wichelhaus

**Affiliations:** ^1^ Department of Orthodontics and Dentofacial Orthopedics LMU University Hospital LMU Munich Munich Germany; ^2^ Inflammatory Origins Murdoch Children's Research Institute Melbourne Australia; ^3^ Melbourne Dental School Faculty of Medicine Dentistry and Health Sciences The University of Melbourne Melbourne Australia

**Keywords:** artificial intelligence, cephalometry, education, landmark identification, orthodontics

## Abstract

This study aimed to evaluate the effectiveness and acceptance of an AI‐supported teaching tool for cephalometric tracing in orthodontic education. Munich Cephalometric Application for Training (MCAT) was introduced to students from two consecutive semesters. Participants were given access to independently train on their own computers. Finally, they submitted the results of three manually traced radiographs. To evaluate the effectiveness, their measurements were compared with those of students from another semester who did not have access to the application. The acceptance was assessed using a questionnaire. Students who used MCAT demonstrated reduced variability in their tracings, with a lower interquartile range compared to the control group. Significant improvements were noted for specific cephalometric variables. The tool was positively received, with 86.5% of the participants perceiving greater learning outcomes for themselves when working with MCAT. MCAT effectively enhances cephalometric tracing skills and is well‐accepted by students, supporting its integration into orthodontic curricula.

## 1 Introduction

Artificial intelligence (AI) is increasingly applied in various aspects of dental diagnosis, treatment planning, and therapy [1, 2, 3]. Future dentists are therefore expected to be proficient in a variety of computer‐assisted tools [4, 5, 6, 7, 8]. Consequently, undergraduate education faces the challenge of providing technology‐enhanced curricula that enables students to acquire fundamental skills and practical experience for daily practice [9, 10, 11]. At the same time, the digital revolution in education [12] has introduced concepts such as e‐learning and blended learning, the benefits of which have been validated by a rising number of studies [13, 14, 15, 16, 17, 18]. Moreover, the COVID‐19 pandemic has transformed dental education globally by increasing the need for implementation of digital teaching formats and online learning opportunities [19, 20, 21]. Recently, the new approbation regulation for dentists in Germany also stipulated that dental students should be familiarized with digital procedures at an early stage in their education [22]. In this regard, the German Medical Faculty Association has pointed out that the developments in the field of artificial intelligence, which will fundamentally change diagnostics in the coming decades, should be taken into account [23].

In undergraduate orthodontic education, the diagnosis of craniofacial morphology using lateral cephalometric radiographs is one of the main focuses of the curriculum. Based upon skeletal and dental anatomical landmarks and their correlations, cephalometric analysis provides information regarding the type and severity of a patient's malocclusion [24, 25, 26]. Dentistry students are currently trained to consistently and precisely identify these anatomical landmarks using the manual tracing method on acetate paper. However, in contemporary orthodontic practice across various regions, this method has been largely superseded by other techniques for cephalometric analysis, notably digital tracing and analysis methods. Recently, automatic landmark identification has gained increased recognition through a rising number of studies highlighting the promising potential of artificial intelligence, especially in the form of deep‐learning models [27, 28, 29, 30, 31]. There are currently several cephalometric platforms available from various commercial vendors (e.g., OneCeph, Hyderabad, India; CellmatIQ, Hamburg, Germany; WebCeph, Republic of Korea; AudaxCeph, Ljubljana, Slovenia) that offer AI‐assisted analysis. However, recent studies assessing the accuracy of commercial frameworks demonstrate that some do not adequately disclose the scientific basis of their AI and are susceptible to inconsistent measurements [32, 33, 34]. Furthermore, AI regulatory policies are still missing and there is a demand to raise awareness and provide the undergraduate dentist with knowledge and practical experiences of the technical changes, advances in the field, and their limitations [35, 36, 37, 38, 39]. Students have a generally positive attitude toward digital applications such as AI‐supported cephalometric tools [40], but there are currently no studies assessing the acceptance and effectiveness of such applications in orthodontic education. For this reason, an independent, non‐commercial AI‐supported framework investigated in a previous study was modified for educational use [41]. Munich Cephalometry Application for Training (MCAT) aims to introduce state‐of‐the art diagnostic measures into undergraduate orthodontic curricula and to ensure the quality of education within the new approbation regulation for dentists in Germany [22, 23]. Upon implementation of an AI‐supported digital education tool for computer‐assisted tracing of cephalometric landmarks, educational institutions can ensure an apprehensive use of AI technology and an integrative development of digital competencies in orthodontic curricula. MCAT encourages the concepts of independent learning and self‐organization by allowing students to progress at their own pace and focus on their difficulties [42, 43]. Such innovative teaching approaches facilitating a learner‐centered approach have the potential to transform education and accelerate progress toward Sustainable Development Goal 4 (SDG 4 ‐ Quality Education) [44, 45].

Yet, it is crucial that AI education tools are critically assessed for their risks and benefits [46]. Hence, in accordance with point 15 of the Beijing Consensus on Artificial Intelligence and Education for educational purposes and assessment [47], MCAT was introduced as part of a pilot test study. The objective of this study was to evaluate the effectiveness and acceptance of MCAT among students.

## 2 Materials and Methods

### 2.1 Study Design

This study was conducted at the LMU University Hospital, Department of Orthodontics and Dentofacial Orthopedics with the approval of the Ethics Committee (Ref. No. 19‐863, 23‐0874 KB) in the final year of the orthodontic undergraduate education. The primary emphasis of this course is to enhance the students' clinical skills in the diagnosis and assessment of malocclusions as well as the formulation of treatment plans. The curriculum includes lectures on cephalometric analysis and orthodontic treatment planning, complemented by division into small seminar groups for detailed case planning. In these seminars, students must perform an in‐depth analysis of provided patient records and devise comprehensive treatment plans for several individual cases, which are subsequently reviewed with lecturers. For the end‐of‐course assessment, students are required to submit the analysis results of three independent cephalometric radiographs traced on acetate paper.

**FIGURE 1:**
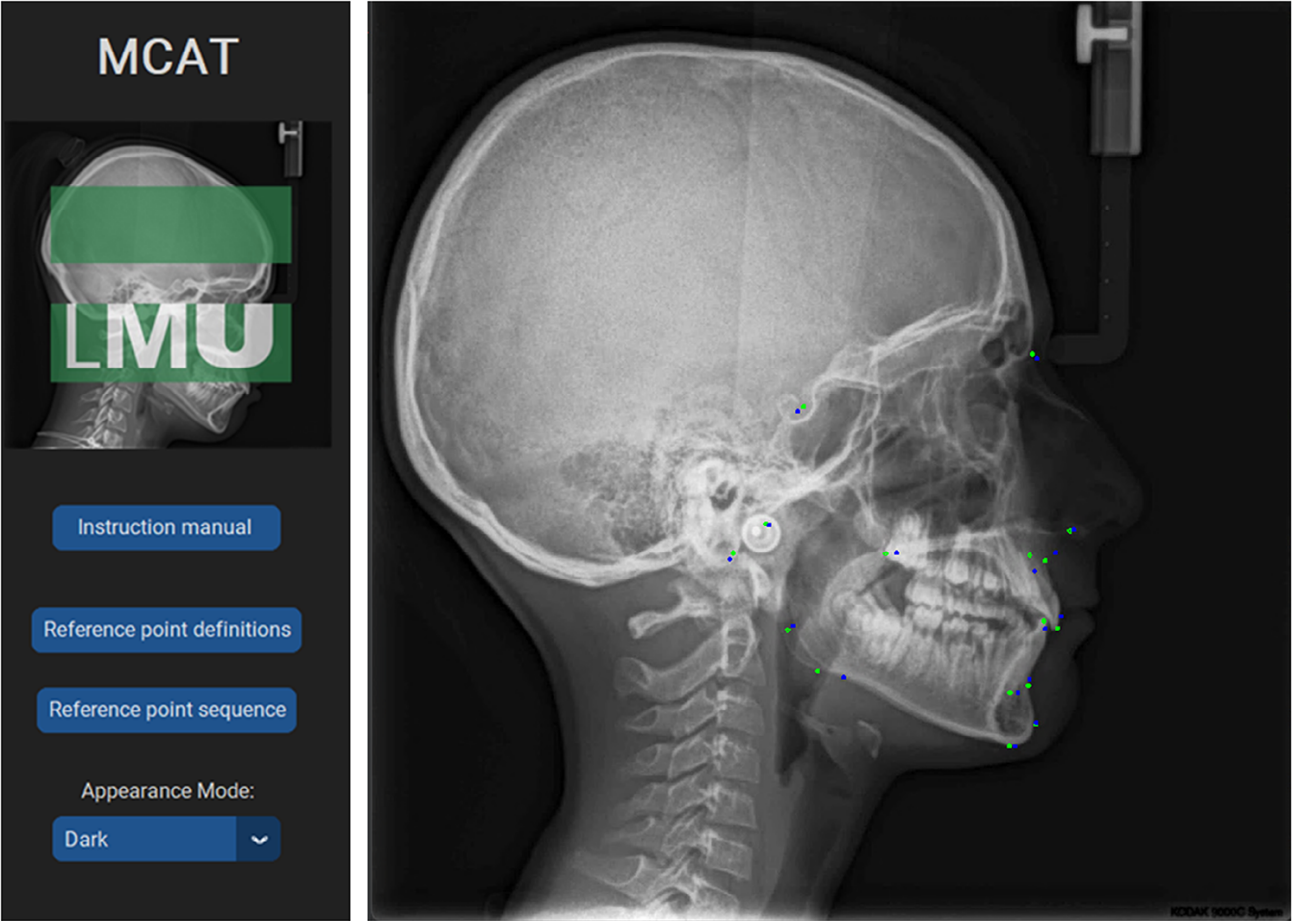
Navigation menu and visual representation of the deviations of the student assessment (blue dots) from the reference standard (green dots) using the MCAT application.

**FIGURE 2:**
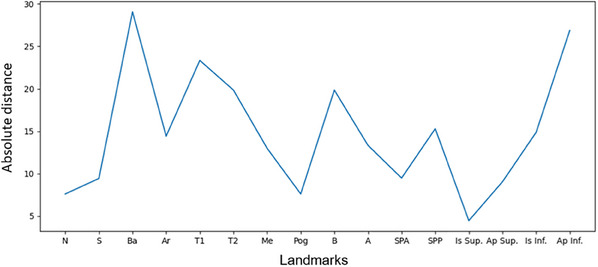
Quantitative representation of the deviations of the student assessment from the reference standard by the MCAT application.

For the present study, this data were collected and analyzed to evaluate the effectiveness of the training in three consecutive semesters. Semester 1 (*N* = 47) was considered the control group that received the regular training protocol, while Semester 2 (*N* = 62) and Semester 3 (*N* = 41) had additional access to an AI‐supported teaching tool (Munich Cephalometric Application for Training [MCAT]) for cephalometric tracing. The introduction of MCAT included a 2‐h training session, during which students received guidance from two orthodontic residents in their final year and one senior orthodontist with over 5 years of experience. Following the on‐site training at the university, the students had the opportunity to access the application for 2 weeks from their personal computers, allowing them to train remotely from any location. The MCAT included a predetermined set of patient cases from which students could manually select a lateral radiograph, trace the cephalometric landmarks, and then review the reference points detected by the automated model (Figure 1). These were displayed in a different colors on the same image for visual reference. In addition, to further assess their performance, participants were provided with a diagram showing the absolute difference between their references and the landmarks detected by the model (Figure 2).

### 2.2 Data

Radiographs were obtained from the archives of the Department of Orthodontics and Dentofacial Orthopedics, University Hospital, LMU Munich. Patient cases with various dentoskeletal morphologies were provided in MCAT as follows: Skeletal Class I, Skeletal Class III, Skeletal Class II, Open Bite, and Deep Bite. Two cephalograms representing each of the above craniofacial anomalies were selected by faculty members so that a total of 10 images were available for training.

Students from Semester 1 submitted three manually traced radiographs after receiving the standard education in the orthodontic study program. Upon completing a training session and access for 2 weeks to MCAT, students in Semesters 2 and 3 also submitted the three manually traced radiographs. Subsequently, the acceptance of the AI educational tool was assessed based on the results from an anonymous questionnaire, while the educational effectiveness of MCAT was determined on the results from the cephalometric analysis on conventional radiographs. The results obtained were digitized and imported into an Excel file (Microsoft Excel for Office 365, Version 16.60, Microsoft Corporation, Redmond, WA, USA) for statistical analysis.

### 2.3 Application

Programming was carried out in Python (Python version 3.11, Python Software Foundation, Beaverton, USA) accessing a pre‐trained model for cephalometric landmark recognition developed in a previous study [41]. The model was trained in advance using a convolutional neural network architecture with 430 cephalometric radiographs in the training dataset. During training pairs of *x*, *y* coordinates of the landmarks and the corresponding cephalometric images were given as input. The underlying data for the pre‐trained model included radiographs of patients with permanent and mixed dentition and did not exclude patients with fixed orthodontic appliances, bands, or brackets. The used model previously achieved an average successful landmark detection rate of 84.73% at 2 mm range and 96.58% at 4 mm range [41].

The graphical user interface (GUI) developed for MCAT was created using the customtkinter library [48]. MCAT was accessed via the students' personal computers and provided a range of components including teaching materials, orthodontic learning resources, a cephalometric tracking tool, and a questionnaire for evaluation. Using MCAT, students had the opportunity to manually trace 16 cephalometric reference points on a lateral cephalometric image and compare their position with the points predicted by the pre‐trained model [41]. Feedback on the deviation of the point set by the students from the predicted point of the model was possible by visual assessment of the lateral cephalometric radiographs (both sets of points were displayed) and additionally by quantitative evaluation in the form of a diagram illustrating the differences between the two sets of points. The quantitative feedback involved calculating the absolute distance (*d*) between the reference point (by the model) and the point determined (positioned by the student) along the *x*‐axis and correspondingly along the *y*‐axis as follows:

$$d=\sqrt{{\left(x_{2}-x_{1}\right)}^{2}+{\left(y^{2}-y^{1}\right)}^{2}}$$

The absolute distance served as feedback for the students (see Figure 1) and helped to evaluate the average error in the definition of the points in the training runs of the students. Calculations were performed using pandas library [49], and plots were automatically rendered using the Matplotlib package [50] (Figure 2).

### 2.4 Evaluation of Effectiveness

**FIGURE 3:**
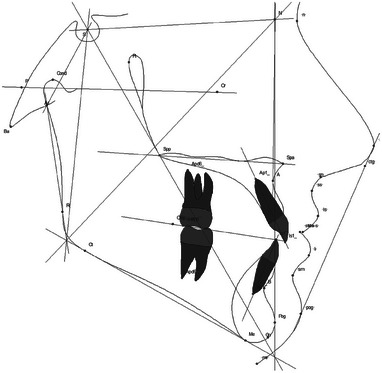
Graphic visualization of cephalometric variables.

For the evaluation of effectiveness, the end‐of‐course assessment tracing results of three independent cephalometric radiographs (Ceph1, Ceph2, Ceph3) were collected for three consecutive semesters (S1, S2, S3). S1 served as the control group, whereas S2 and S3 were designated as intervention groups. The total learning time was identical for all groups. However, the control group received standard training, whereas the intervention groups utilized the AI‐assisted learning tool. The deviations from the department's reference standard were determined. The reference standard was developed by consensus from the lecturers (two senior physicians and two orthodontic residents). Nineteen cephalometric variables were evaluated (Table 1 and Figure 3).

**TABLE 1:** Cephalometric variables evaluated and their definitions (also see Figure 3).

Cephalometric variable	Definition
SNA (°)	Angle between Sella (S), Nasion (N), and Subspinale (A); anteroposterior position of the maxilla relative to the cranial base
SNB (°)	Angle between Sella (S), Nasion (N), and Supramentale (B); anteroposterior position of the mandible relative to the cranial base
SNPg (°)	Angle between Sella (S), Nasion (N), and Pogonion (Pog); anteroposterior position of the chin relative to the cranial base
ANB (°)	Angle between Subspinale (A), Nasion (N), and Supramentale (B); anteroposterior relationship between the maxilla and mandible
NSBa (°)	Angle between Sella (S), Nasion (N), and Basion (Ba); cranial base angle
ML‐NSL (°)	Angle between the Mandibular Line (ML) and Nasion‐Sella Line (NSL); inclination of the mandibular plane relative to the cranial base
NL‐NSL (°)	Angle between the Nasal Line (NL) and Nasion‐Sella Line (NSL); inclination of the maxillary plane relative to the cranial base
ML‐NL (°)	Angle between the Mandibular Line (ML) and Nasal Line (NL); relative inclination of the mandibular to the maxillary plane
ArGoMe (°)	Angle between Articulare (Ar), Gonion (Go), and Menton (Me); gonial angle
ArGoN (°)	Angle between Articulare (Ar), Gonion (Go), and Nasion (N); upper gonial angle
MeGoN (°)	Angle between Menton (Me), Gonion (Go), and Nasion (N); lower gonial angle
NSp/SpME × 100 (%)	Ratio of the distance from Nasion to Spine (NSp) and Spine to Menton (SpMe), expressed as a percentage; anterior facial height
SGo/NMe × 100 (%)	Ratio of the distance from Sella to Gonion (SGo) and Nasion to Menton (NMe), expressed as a percentage; relation between anterior and posterior facial height
U1‐NL (°)	Angle between the upper incisor (U1) and the Nasal Line (NL); inclination of the upper incisors relative to the maxillary plane
U1‐NA (mm)	Distance between the tip of the upper incisor (U1) and a perpendicular line from point A (NA); anteroposterior position of the upper incisors
L1‐ML (°)	Angle between the lower incisor (L1) and the Mandibular Line (ML); inclination of the lower incisors relative to the mandibular plane
L1‐NB (mm)	Distance between the tip of the lower incisor (L1) and a perpendicular line from point B (NB); anteroposterior position of the lower incisors
U1‐L1 (°)	Angle between the upper incisor (U1) and lower incisor (L1); the interincisal angle
Wits (mm)	Occlusal plane's intersection points with the A and B points; anteroposterior jaw relationship
Harvold (mm)	Difference between the distances Condylion (Cond) and Pogonion (Pog) and Cond and Subspinale (A); maxillomandibular difference

### 2.5 Evaluation of Acceptance

A questionnaire was developed to determine the acceptance of MCAT. This was completed anonymously via the educational platform “Moodle” (Modular Object‐Oriented Dynamic Learning Environment, West Perth WA 6872, Australia) by the students of Semesters 2 and 3. To create the questionnaire, literature research was conducted using the keywords “digital learning,” “Likert Scale,” “remote learning,” and “digital cephalometry.” This generated 52 questions, which were ranked by the authors using the nominal group technique (NGT) [51]. The final version of the questionnaire was prepared with 26 questions, taking into account a maximum processing time of 15 min. Most of the answer options were based on a modified Likert scale [52, 53]. In addition, multiple answer options and free‐text responses were also offered. The results were statistically analyzed, whereby the two most positive answer options (e.g., “very good” and “good”) were combined into “positive agreement.”

### 2.6 Statistical Analysis

All analyses were performed using R Statistical Software (Version R‐4.3.3., R Development Core Team, Vienna, Austria) [54]. The agreement between students’ ratings and the reference (gold standard) was assessed with an intraclass correlation coefficient (ICC), using Pearson's correlation coefficient, and a “pairwise complete observation” method for computing covariances in the presence of missing values [55]. The “cor” function, as a part of base R, was used to calculate ICC, and the corresponding significant testing confirmed with CCC function of epiR package [56].

**FIGURE 4:**
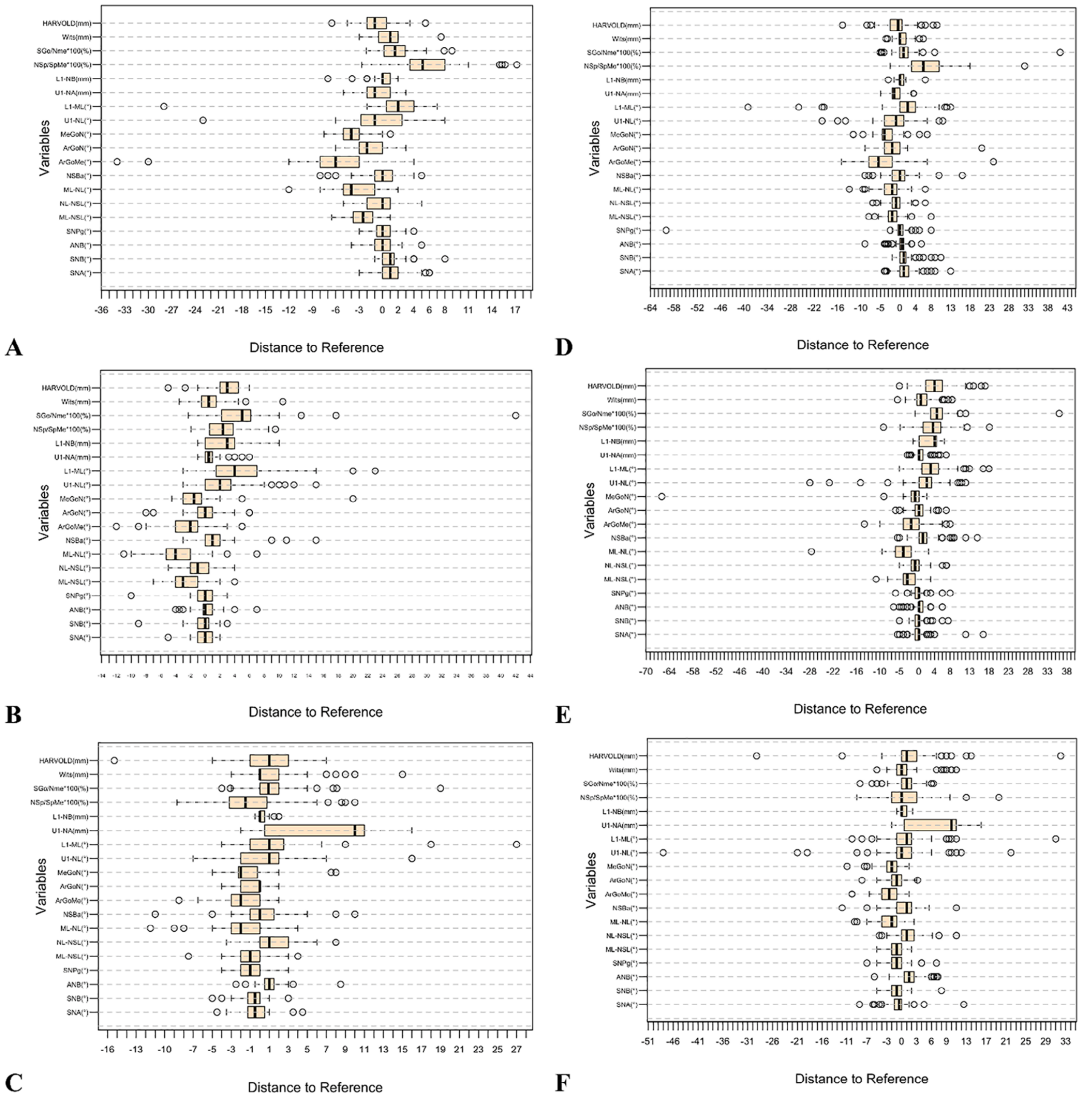
Deviation of students’ point selection compared to the reference model. (A–C) Control group (Semester 1); (D–F) summary of Semester 2 and Semester 3; (A and D) Ceph1; (B and E) Ceph2; (C and F) Ceph3.

Recordings of 19 variables, submitted by students in Semesters 1 (control group), 2 and 3 (intervention groups) for Ceph1, Cepth2, and Ceph3 were collected in an Excel file. The difference between the average of each variable for all the student and the reference value, alongside their respective standard deviations were calculated for Ceph1, Ceph2, Ceph3 for each semester. To evaluate the differences between the conventional evaluation method and the MCAT‐based learning method, we compared Semester 1 scores (conventional method) with pooled scores from Semesters 2 and 3 (MCAT‐based method). The Shapiro–Wilk test was used to assess the normality of the scores for both the conventional and MCAT‐based methods. As the scores in both groups were not normally distributed, the Mann–Whitney *U* test (Wilcoxon rank‐sum test) was employed to compare the scores between the conventional method and the MCAT‐based method. The analysis was conducted for all measured variables (Ceph1, Cepth2, and Ceph3). Deviation of students’ measurements from the reference, separately for control and intervention semesters was plotted for all 19 variables (Figure 4).

Agreement between the average of each variable for all the student and the reference values was assessed using the Bland–Altman analysis, also known as a difference plot, which plots the differences between the measurements against their averages. The limits of agreement were calculated as the mean difference ± 1.96 standard deviations of the differences, which is expected to contain 95% of the difference between the two methods. The BlandAltmanLeh, psych, ggplot2, ggExtra, and ggrepel packages in R were utilized for generating Bland–Altman plots and calculating limits of agreement [57, 58, 59, 60]. Statistical significance was set at a *p*‐value less than 0.05.

## 3 Results

### 3.1 Effectiveness

Students from two semesters with assisted learning using MCAT (intervention groups S2 and S3) were compared with students who only learned using the conventional method (control group S1). Both were evaluated on three independent cephalometric images (Ceph1, Ceph2, and Ceph3) based on the difference from the reference standards. The mean deviations between the students tracing results and the reference standard are shown in Table 2.

**TABLE 2:** Deviations between the students tracing results of Semester 1 (S1), Semester 2 (S2), Semester 3 (S3) and the three cephalometric images (Ceph1, Ceph2, Ceph3) to the reference standard.

Variable	Ceph1						*p*‐values	Ceph2						*p*‐values	Ceph3						*p*‐values
	S1 (*N* = 47)		S2 (*N* = 62)		S3 (*N* = 41)			S1 (*N* = 47)		S2 (*N* = 62)		S3 (*N* = 41)			S1 (*N* = 47)		S2 (*N* = 62)		S3 (*N* = 41)		
	Mean	SD	Mean	SD	Mean	SD		Mean	SD	Mean	SD	Mean	SD		Mean	SD	Mean	SD	Mean	SD	
**SNA (°)**	−1.18	2.11	−1.50	2.15	−0.75	3.02	*0.963*	−0.02	1.29	0.21	1.65	0.00	3.80	*0.084*	0.51	1.48	0.69	1.46	1.25	2.93	*0.112*
**SNB (°)**	−1.19	1.56	−1.27	1.59	−1.28	2.23	*0.833*	0.18	1.70	0.48	0.87	−0.03	2.10	*0.060*	0.69	1.42	1.03	1.08	0.94	2.11	*0.081*
**ANB (°)**	0.16	1.85	−0.43	1.53	0.25	2.25	*0.219*	−0.27	1.77	−0.55	1.40	−0.02	2.37	*0.507*	−1.10	1.51	−1.90	2.02	−1.54	2.61	*0.160*
**SNPg (°)**	−0.22	1.32	0.71	7.74	−0.29	1.74	*0.808*	0.09	1.85	0.38	1.03	−0.26	2.33	*0.114*	0.81	1.40	1.08	1.55	0.82	2.14	*0.265*
**ML‐NSL (°)**	2.43	1.86	1.76	2.06	1.66	2.19	***0.034***	2.41	2.26	2.73	1.65	2.38	2.67	*0.886*	1.03	1.93	1.40	1.43	0.92	1.67	*0.623*
**NL‐NSL (°)**	0.18	2.20	1.14	2.25	0.87	2.36	*0.053*	0.83	1.96	0.91	1.81	0.68	2.44	*0.979*	−1.63	2.60	−1.45	2.34	−1.33	2.67	*0.628*
**ML‐NL (°)**	3.47	2.86	2.79	2.57	2.10	3.39	*0.053*	3.68	3.30	4.53	3.96	3.19	2.76	*0.787*	2.00	2.82	2.47	2.30	2.08	2.33	*0.258*
**NSBa (°)**	0.21	2.73	0.21	2.65	0.10	3.83	*0.645*	−1.56	3.09	−1.92	3.30	−1.37	2.51	*0.591*	−0.48	3.11	−0.60	2.61	−0.82	3.09	*0.374*
**ArGoMe (°)**	6.02	6.66	5.40	3.73	4.28	6.08	*0.960*	2.32	3.32	2.03	2.79	2.02	4.01	*0.649*	2.04	2.27	2.84	2.07	2.61	2.19	*0.055*
**ArGoN (°)**	1.43	2.02	2.18	2.23	1.53	4.26	*0.135*	0.15	2.41	0.18	1.57	−0.18	2.27	*0.939*	0.80	1.50	1.35	1.62	0.39	1.61	*0.351*
**MeGoN (°)**	3.62	1.97	3.31	2.59	3.43	2.64	*0.460*	1.05	3.59	2.42	8.29	1.08	1.88	*0.218*	1.17	2.41	1.88	1.35	2.42	2.45	*0.126*
**U1 – NL (°)**	0.48	5.06	0.94	3.82	1.57	5.30	*0.375*	−2.46	3.98	−1.78	4.84	−0.70	5.41	*0.901*	−1.04	4.30	−0.06	7.23	−0.36	6.21	*0.804*
**L1 – ML (°)**	−1.60	4.85	−1.78	6.94	−0.91	6.17	*0.743*	−4.84	5.39	−4.02	4.30	−2.47	3.57	*0.084*	−1.83	5.16	−1.29	4.99	−0.50	3.38	*0.469*
**U1‐ NA (mm)**	0.94	1.87	1.24	1.50	0.81	2.03	*0.638*	−0.91	1.64	−0.24	1.28	−0.95	1.81	*0.191*	−6.85	5.74	−7.46	5.05	−7.32	5.26	*0.972*
**L1 – NB (mm)**	0.10	1.50	−0.36	1.07	−0.31	0.75	*0.208*	−2.40	2.72	−2.55	2.26	−2.34	2.42	*0.573*	−0.31	0.56	−0.44	0.63	−0.34	0.63	*0.416*
**NSp/SpMe x 100 (%)**	−6.02	4.43	−6.97	5.73	−6.23	5.02	*0.444*	−2.72	2.58	−3.97	3.94	−2.64	3.83	*0.166*	0.65	4.65	−0.61	4.39	−0.75	4.23	***0.035***
**SGo/NMe x 100 (%)**	−1.72	2.17	−0.73	2.05	−1.86	6.73	***0.043***	−5.33	6.60	−4.95	4.65	−4.43	2.86	*0.961*	−1.48	3.48	−0.85	2.29	−0.81	2.27	*0.993*
**Wits (mm)**	−0.54	1.77	−0.53	1.48	−0.61	1.91	*0.947*	−0.95	2.13	−1.02	2.20	−0.95	2.96	*0.767*	−1.70	3.74	−0.73	2.93	−1.33	3.93	*0.134*
**HARVOLD (mm)**	0.65	2.47	0.48	3.17	1.25	3.13	*0.967*	−2.67	2.36	−3.77	4.04	−4.15	3.54	*0.127*	−0.69	3.88	−1.79	6.64	−2.03	3.62	*0.202*

*Note*: The calculation of *p*‐values was based on the Mann–Whitney *U* test (Wilcoxon rank‐sum test).

The results showed that deviations were not uniform across all landmarks. Some landmarks exhibited higher deviations, while others showed mean deviations close to 0°. For instance, the variables ML‐NL and ArGoMe had increased deviations, ranging from 2.00° to 6.02° across all cephalometric images (Ceph1, Ceph2, and Ceph3). Other variables showed less consistent deviations when comparing the outcomes of the three individual tracings. For example, MeGoN showed values consistently above 3°, while U1‐NA exhibited increased deviations around 7° only in Ceph3 in contrast to the results in Ceph1 and Ceph 2 with values of 0.94 and −0.91, respectively.

Other variables such as SNA, ANB, NL‐NSL, NSBa, U1‐NL, U1‐NA, L1‐NB, and Wits frequently showed low deviations below 2° or 2 mm.

Significant differences between the control (S1) and intervention groups (S2 and S3) were observed in specific landmarks such as ML‐NSL and SGo/NMe for Ceph1 and NSp/SpMe for Ceph3.

The boxplots in Figure 4 provide a detailed graphical evaluation of the data. Figure 4A–C illustrates the control group (S1), while Figure 4D–F presents the pooled data from the intervention groups (S2 and S3). Outliers are identified in both groups across all variables. The control group (S1) exhibited a higher interquartile range compared to the intervention groups (S2 and S3). The variable U1‐NA showed a comparatively high deviation and large interquartile range at Ceph3 (Figure 4C,F), which was not observed in the other cephalometric images.

### 3.2 Acceptance

**FIGURE 5:**
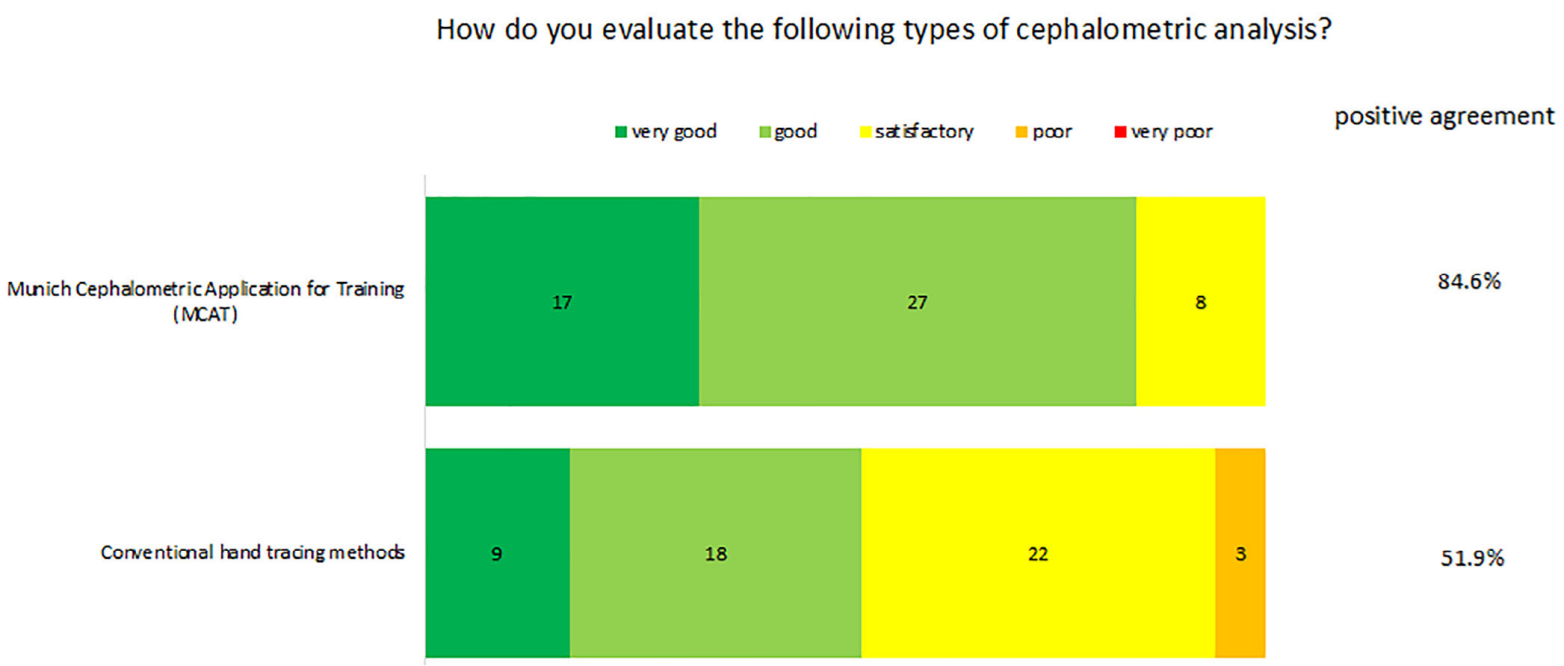
Students assessment of MCAT in comparison to conventional manual trading methods. Rating was conducted using a modified Likert scale (1 = very good, 2 = good, 3 = satisfactory, 4 = poor, 5 = very poor). Given are the two formats (left) and the absolute number of given answers (middle). In addition, ratings 1 and 2 are summarized together as positive agreement (right).

The acceptance of the AI‐supported teaching method among the students was also determined and evaluated using a questionnaire with an underlying modified Likert scale. The results are illustrated in Figure 5.

The students of the final course had almost no experience in digital cephalometry, let alone with AI‐supported cephalometric teaching (96.2%); 48% of the participants worked with a Mac and 50% with a Windows computer, 1.9% used other hardware configurations. Students rated the digital tracing of a cephalometric radiograph with the help of MCAT very positively (positive agreement 84.9%). In this context, the manual tracing was rated with 51.9% (Figure 5). In a direct comparison, 86.5% of the participants saw greater learning success for themselves when working with MCAT. The participants were particularly impressed by the large number of radiographs (94.2%), the fast and simple function of the program (88.5%), the immediate feedback (78.8%), and the linking of theoretical and practical knowledge (84.6%).

Interestingly, the students in the study felt that they had not been sufficiently taught in cephalometry analysis in their previous academic studies (28.8%). The participants criticized problems with the opening of MCAT (32.7%). Only 63.5% of the students considered themselves to have a high learning efficiency due to the close supervision on site. In summary, 94.2% of the participants were in favor of a long‐term implementation of MCAT in the orthodontic curriculum.

## 4 Discussion

This study aimed to evaluate the effectiveness and acceptance of an open‐source, AI‐supported teaching tool for undergraduate education in orthodontic cephalometric analysis.

Cephalometric analysis is essential for orthodontic diagnosis, treatment planning, and treatment evaluation, and is an integral part of the dental curriculum according to the German approbation regulation for dentists, as well as other undergraduate and postgraduate curricula. While digital tools and AI‐supported methods are becoming increasingly prevalent, manual cephalometric tracing remains a core component of orthodontic training in most regions, including Europe and the United States [61, 62, 63]. However, limited on‐site education time and human resources pose significant challenges in the educational system. Studies have found that students' overall accuracy in landmark identification is low, while the required tracing time is comparatively high [64]. To address this challenge, several research groups proposed the use of digital and web‐based applications for educational purposes, using desktop computers or touch‐input devices such as tablets and smartphones [63, 64, 65].

In the present study, a software program was developed based on a previously evaluated AI model [41]. The utilization of an AI model allows for the efficient provision of a large number of images and the use of a validated reference standard, which can be advantageous over conventional digital training tools. MCAT allows students to train at their own pace and receive both qualitative and quantitative feedback on their performance. The authors chose to evaluate the effectiveness of MCAT concerning its impact on the legally required manual tracings, as the primary goal of the teaching tool is to enhance students' preparation for and performance in examinations.

Acceptable deviations in cephalometric analysis are usually set within 2 mm or 2°, which is considered clinically insignificant and within the margin of error for manual tracings [65, 66]. However, certain landmarks are more challenging to identify and typically show comparatively higher deviations, while others show higher deviations due to the wider variation of their anatomical localization [67]. For instance, landmarks placed on anatomically formed edges are easier to identify, whereas landmarks placed on curves are more prone to error [61]. Finally, the clinical significance of a deviation varies between landmarks, depending on the range in which the diagnosis changes regarding the respective analysis.

The results of this study showed that the mean deviations between the intervention (S2 and S3) and control (S1) groups did not differ significantly, except for the variables ML‐NSL and SGo/NMe (Ceph1), and NSp/SpMe (Ceph3). The fact that the statistically significant differences only occurred in one respective image suggests that they are rather negligible. Notably, specific variables such as ML‐NL and ArGoMe showed higher deviations, indicating areas where students might need more targeted training. This is in line with previous findings, highlighting that the gonion and lower incisor apex are the least consistent landmarks [68]. Overall, the mean deviations for most variables were comparatively low for both, the intervention and control groups, compared to the outcomes of other studies that reported student outcomes [64]. The high observed educational skill level, already evident in the control group, may have contributed to the limited number of significant differences. However, in both groups, outliers were frequent and generally a high standard deviation was observed.

Although most differences were not statistically significant, the lower interquartile range in the intervention groups (S2 and S3) implies that the AI‐supported tool helped standardize the learning process, making students' performance more uniform. This supports previous findings that digital tools can enhance learning outcomes by providing consistent and immediate feedback.

With regard to the acceptance of the teaching tool, students rated the digital tracing of cephalometric radiographs using MCAT very positively (positive agreement 84.9%). In contrast, manual tracing was only positively evaluated by 51.9% (Figure 5). In particular, the possibility of training on a large number of radiographs in MCAT was positively highlighted (94.2%). The findings are in line with the results from Alhazmi et al., who reported that 92.7% preferred digital over manual tracing technique [62]. Learning is a repetitive process characterized by applying and repeating [69]. At the same time, the participants in the study felt that the linking of theoretical and practical learning was particularly effective (positive agreement: 84.6%). In favor of the two points mentioned above is that it is important to have a routine within the cephalometric analysis [70]. Consistently with this, the study participants valued the direct and instant feedback (78.8%). Each student could immediately view the correct answer or reference and see the deviation from their given answer. Some students also praised the various digital setting parameters, such as exposition time, contrast settings, as well as the zoom function, as these possibilities would not be available in manual tracing. Only 63.5% of the participants saw a high learning efficiency for themselves due to the close supervision on site. The authors consider this positive, as such time‐intensive support as in the pilot study is not planned for the future. MCAT is designed as a remote learning model independent of location [71].

When discussing the acceptance of the teaching tool MCAT, limitations must also be mentioned, such as the novelty effect [72]. An innovation or a new learning program may be evaluated better with its introduction than it is actually perceived. Therefore, the result for manual tracing, without the offer of the MCAT, might have turned out better. For the design of a crisis‐proof teaching, as well as the possibility of remote learning, location‐independent working with simultaneous compatibility with all common operating systems must be guaranteed. According to Asiry, access to teaching media is a precondition for successful digital learning [73]. Our results show that Mac and Windows computers were used equally. Most problems occurred when opening the program (32.7%). It should be noted that the 10th, final, semester took part in this study. This could be a reason for the predominantly positive evaluation of MCAT, because some participants saw the program as a special opportunity for additional exam preparation. No participant criticized a time‐consuming familiarization with the new program. On the contrary, many participants valued the quick and easy functioning of the program (88.5%). Another reason for the good acceptance of MCAT could be that the participants felt that they had not been taught enough in the area of cephalometric teaching (positive agreement 28.8%)—even though they had already been taught to draw a cephalometric radiograph by hand. Finally, it is not surprising that the students would like to see MCAT implemented in the orthodontic curriculum in the future (94.2%). This is particularly necessary with regard to progressive teaching and to meet the requirements of the new licensing regulations in Germany [22].

Several limitations must be acknowledged. The study was conducted at a single institution, which may limit the generalizability of the findings. When interpreting the effectiveness of MCAT, it should be considered that students received additional training using a digital tracing method, whereas the evaluation of the end‐of‐course assessments was based on manual tracing evaluations. The authors consider this approach to be appropriate due to the legal regulations and requirements of the curriculum, as the aim of developing MCAT was to improve the prerequisites for the examination. However, as a result, the effect of the teaching tool training on cephalometric skills may have been detectable only to a lesser extent. It should be noted that both the intervention and control groups had the same amount of learning time but employed different methods.

Another difficulty and possible source of bias is the reference standards used. In general, the issue of defining a universal standard remains unresolved for both manual and digital tracing methods. The reference standards used in this study were developed by expert consensus, which is the currently accepted practice, but may be subject to bias. MCAT was developed based on a pre‐trained AI model, which achieved between 84.73% and 96.58% accuracy depending on the search radius. This implies that there is a certain residual risk that a student's correct anatomical annotation will be classified as deviant. However, the visual representation in the software enables students to check and assess the corresponding landmarks independently. The annotations for the exercise images were also reviewed by the instructors prior to the study. Finally, the time students trained with MCAT was not assessed, which could affect the effectiveness of the teaching tool.

Future research could investigate the effect of cephalometric teaching tools in a multicenter approach and compare different training methods and cephalometric evaluation models within a larger cohort. Furthermore, longitudinal studies could evaluate student's performance over a longer time period to assess the sustainability of their skills.

The positive reception of MCAT by the students underscores its potential as a valuable addition to orthodontic education. The AI‐supported tool helped standardize the learning process, making students' performance more uniform. The ability to provide immediate feedback and allow independent, self‐paced learning aligns well with contemporary educational approaches emphasizing digital competence and self‐directed learning. Given the new approbation regulations in Germany, integrating tools like MCAT may help to ensure that dental students are well‐prepared for modern clinical practice.

## 5 Conclusions

The teaching tool MCAT was positively received among students, with 84.9% agreeing that AI‐supported teaching was helpful, 86.5% stating that they achieved greater learning success with MCAT, and 94.2% in favor of long‐term implementation.

The evaluation of the effectiveness of MCAT showed significant differences between AI‐assisted learning and conventional learning for individual landmarks in the different cephalometric images, and its use resulted in a generally reduced variability in examination tracings.

## Author Contributions

HS: study design, methodology, investigation, data interpretation, manuscript (original draft preparation). TP: data collection, software, manuscript (original draft preparation). LvB: data collection, questionnaire, manuscript (original draft preparation). LH: data curation, manuscript (original draft preparation). YK: data analysis and statistical analysis, manuscript (original draft preparation). AW: project administration, supervision, manuscript (review and editing).

## Conflicts of Interest

The authors declare no conflicts of interest.

## Data Availability

The teaching tool MCAT was published online and is available at Open Data LMU (https://data.ub.uni‐muenchen.de/). The datasets and codes generated and analyzed are available from the corresponding author upon reasonable request.
